# Preparing for the Next Pandemic: Learning From COVID-19 to Build What Comes Next

**DOI:** 10.1093/ofid/ofag348

**Published:** 2026-07-10

**Authors:** Maria Van Kerkhove

**Affiliations:** Acting Director of the Department of Epidemic and Pandemic Management, Health Emergencies Programme, World Health Organization, Geneva, Switzerland

**Keywords:** epidemic, pandemic, preparedness, COVID-19, Ebola, Hanta

## Abstract

WHO's efforts to strengthen pandemic preparedness—grounded in what the world learned during COVID-19 and what today's outbreaks of avian influenza, Hantavirus and Ebola are teaching us.

Respiratory viruses remain among the most persistent and consequential global health threats. They spread efficiently, evolve rapidly, and can overwhelm health systems before the full scope of their impact is recognized. The COVID-19 pandemic—the most significant global health crisis of our generation—is now in its postacute phase, but the conditions that enabled SARS-CoV-2 to emerge and spread with such devastating effect have not changed enough. The question before us is not whether another pandemic-capable respiratory virus will emerge, but whether we will be ready when it does.

WHO works with Member States and partners every day to assess signals from influenza and coronavirus surveillance, novel respiratory pathogen detections, and outbreaks that may not yet be in the headlines. While some events remain localized, others carry the potential to expand across borders. Preparedness is the work of ensuring that when the next serious threat appears, the global community is faster—faster to detect, faster to share information more effectively, communicate with communities more efficiently, protect those at highest risk more quickly, and scale countermeasures equitably.

COVID-19 cost lives, livelihoods, and trust—but it also taught lessons we cannot afford to forget. This commentary reflects that vantage point: what COVID-19 taught us and how those lessons are actively shaping WHO's work with Member States and partners to prepare for the next epidemic and pandemic threat.

## WHAT COVID-19 TAUGHT US

COVID-19 accelerated scientific and operational advances at historic speed. Countries expanded surveillance and laboratory capacities, built new data pipelines, and worked to implement public health and social measures while attempting to maintain essential services. The rapid development of diagnostics, therapeutics, and vaccines—supported by decades of prior research and global scientific collaboration [1]—demonstrated what is possible when urgency meets investment, solidarity and shared goals.

The pandemic also exposed deep and longstanding vulnerabilities that predated COVID-19 and were dramatically amplified by it. Inconsistent surveillance and laboratory capacity meant outbreaks were detected late and reported slowly in many settings. Health and care workers faced shortages of personal protective equipment and chronic workforce strain. Inequitable access to diagnostics, oxygen, personal protective equipment, therapeutics, and vaccines prolonged transmission and cost lives that did not need to be lost. In many settings, risk communication failures and the spread of misinformation and disinformation eroded the public trust that is essential to effective response [2]. This is as true for respiratory viruses as it is for other high threat pathogens such as the current Ebola Bundibugyo Virus Disease in the Democratic Republic of the Congo and the Hantavirus outbreak on the MV Hondius cruise ship [3, 4].

Perhaps most consequential was the structural failure of equity. Vaccines were developed in under a year; a historic scientific achievement, yet access concentrated in wealthier nations while variants circulated unchecked in under-resourced settings. These failures reflected the architecture of global health systems of the past that were not designed with equity at their center. The result was a crisis that was longer, more severe, and more unequal than it needed to be.

**Figure 1.**
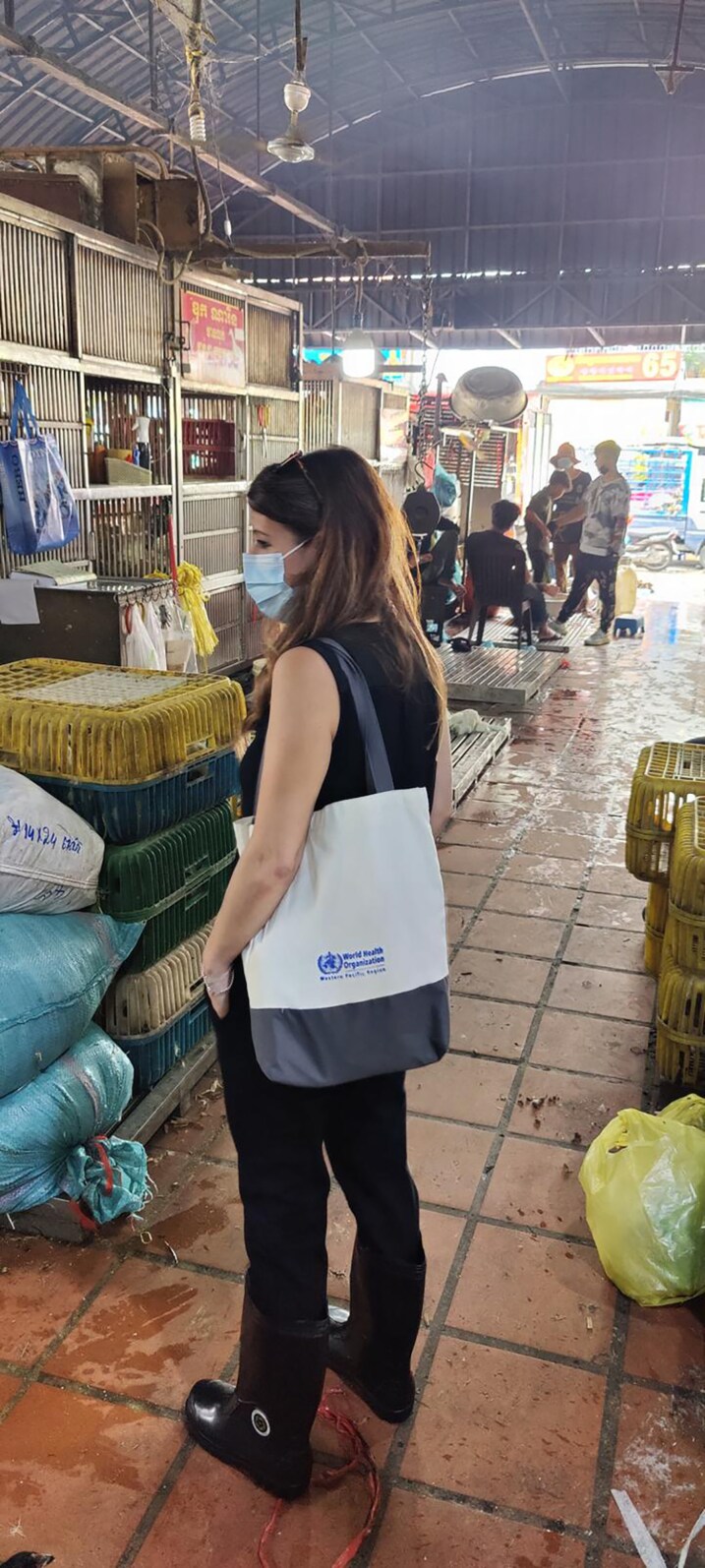
Dr Maria Van Kerkhove, visiting a live animal market in South East Asia.

COVID-19 also demonstrated how political fragmentation and the cycle of panic followed by neglect can slow collective action at precisely the moments when speed is most important. Preparedness cannot depend on emergency appeals and short-term political attention. It has to be embedded in long-term planning, budgets, laws, and routine public health work, with regular testing of plans and capabilities. That lesson, more than any other, defines the work ahead (Figure 1).

## SEVEN LESSONS WE CANNOT FORGET

Looking across the COVID-19 experience and the preparedness work now underway, 7 lessons stand out as most consequential for what comes next:

- Global threats demand global solidarity. Pathogens with epidemic and pandemic potential, especially respiratory viruses, move faster than borders, politics, and reactive cooperation. No country is safe until all countries are safe. Solidarity must be designed into preparedness, not mobilized after the outbreak has already spread.
- Equity must be built in from the start. Inequitable access to vaccines, diagnostics, personal protective equipment, oxygen and therapeutics prolonged the pandemic and cost lives. Equity is not charity—it is a core requirement for health security. Preparedness must include equitable pathways across research, manufacturing, allocation, and delivery from the outset that reach those at highest risk.
- Strong health systems are the foundation of resilience. Countries with strong community-based systems, robust primary care, public health networks and empowered health, and care workers were more resilient. Preparedness starts in everyday investments in workforce, surveillance, laboratories, and trusted services—long before an outbreak.
- Speed, transparency, and trust save lives. Time lost is lives lost. Early detection, timely information-sharing, and clear, consistent risk communication are lifesaving. Systems should reward transparency, not penalize it. Misinformation must be addressed with the same urgency as the pathogen itself, because trust is just as vital as any medical countermeasure.
- We must break the cycle of panic and neglect. In every crisis there is a rush to act—and then a rush to forget. Preparedness cannot survive on emergency attention alone. It must be embedded in long-term planning, budgets, and routine public health work, with regular exercises and accountability.
- Effective governance accelerates action. When roles are unclear and coordination fragmented, response slows. Stronger global governance—anchored in the WHO International Health Regulations (IHR) and reinforced through the WHO pandemic agreement creates clarity and accountability; conditions for faster, collective action [5, 6].
- Preparedness and prevention must embrace One Health. Threats with epidemic and pandemic potential emerge at the intersection of human, animal, and environmental health. Coordinated action across human health, animal health, agriculture, environment, wildlife and climate sectors, before outbreaks begin, is the only way to reduce spillover risk and amplification at its source.

## HOW WHO IS PREPARING FOR THE NEXT PANDEMIC

These lessons are not theoretical. WHO and partners have worked with countries since the first days of the COVID-19 pandemic, throughout the crisis phase and into today's dealings with zoonotic influenza, cholera, dengue, hantavirus, Ebola, and more to translate lessons into practical preparedness capabilities, so that when the next serious threat appears, countries can detect it earlier, respond faster, and protect those at highest risk more equitably. Some examples of applying lessons are below.

Earlier warning through better intelligence is foundational. Improvements in surveillance, including through strengthened laboratory systems, and timely reporting through the IHR is critical as infectious diseases do not respect borders and coordinated global actions are critical [2]. The WHO Hub for Epidemic and Pandemic Intelligence was established in 2021 to expand collaborative data networks for earlier detection and improved analytics, including the Epidemic Intelligence from Open Sources system for event-based surveillance [7, 8]. The Hub, which is part of the WHO Health Emergencies Programme, works to improve how countries detect, monitor, and manage public health threats and to inform decision-making on broader policy issues to mitigate these threats.

Equity in access to countermeasures requires structural solutions, not emergency improvisation. WHO's mRNA technology-transfer program supports local and regional capacity to develop and produce vaccines and other health technologies, directly addressing one of the most consequential failures of the COVID-19 response [9]. The WHO Biomanufacturing Workforce Training Initiative, including a Global Training Hub in the Republic of Korea, supports countries in building the skills needed for sustainable production and quality assurance. The WHO BioHub and the interim Medical Countermeasures Network provide additional pathways for the sharing of information and pathogens and for equitable access to diagnostics, therapeutics, and vaccines during emergencies [10, 11].

Financing core capacities requires sustained commitment. The Pandemic Fund, established by WHO and the World Bank and partners, supports investments in pandemic prevention, preparedness, and response—including in lower-income countries [12]. Surge capacity through the Global Health Emergency Corps provides a framework to strengthen health emergency workforce capacity and collaboration when it matters most [13].

Stronger global governance is essential to converting individual actions into coordinated response. Member States adopted the WHO Pandemic Agreement at the World Health Assembly in May 2025, and amendments to the IHR entered into force in September 2025 [5, 6, 14]. These instruments are designed to strengthen accountability structures, data-sharing obligations, and coordination mechanisms that were lacking at critical moments during COVID-19. Ongoing negotiations toward a pathogen access and benefit sharing system aim to enable faster pathogen sharing alongside more equitable access to the countermeasures that follow—directly addressing the structural asymmetry that shaped the COVID-19 vaccine rollout.

## WHAT NEEDS TO HAPPEN BEFORE THE NEXT PANDEMIC

The next pandemic threat may look different from SARS-CoV-2, but the requirements for readiness are consistent: sustained financing, resilient national public health systems, faster detection and transparent data-sharing, protected health workers, trust in communities, and equitable access to countermeasures. At the core of this must be a commitment to equity and placing it at the center of every decision, from surveillance and research priorities to the allocation and delivery of resources. Science, data, and the benefits they yield must be shared openly, ensuring that early warning translates into early action everywhere, not just in settings with the greatest resources.

Preparedness cannot remain reactive. It requires sustained investment in prevention and readiness as routine public health functions, embedded within laws, budgets, and institutions, and reinforced through regular exercises and accountability [15]. This work must endure through political transitions, competing priorities, and fiscal pressures. It also demands collective action across sectors, including One Health approaches that address the upstream drivers of spillover risk at their source [16]. Framing preparedness as something built only in “peacetime” is itself flawed—because for many communities, the emergencies never fully stop.

COVID-19 tested our systems and our resolve. It showed what happens when we delay, divide, and deprioritize health. But it also demonstrated what is possible when science leads, communities are engaged, and solidarity holds. The goal now is not recovery alone—it is to transform what we learned into everyday readiness, so that when the next pandemic emerges, we respond faster, fairer, and together. That is the legacy COVID-19 demands of us.
